# P-glycoproteins play a role in ivermectin resistance in cyathostomins^☆^

**DOI:** 10.1016/j.ijpddr.2017.10.006

**Published:** 2017-10-25

**Authors:** L.E. Peachey, G.L. Pinchbeck, J.B. Matthews, F.A. Burden, A. Lespine, G. von Samson-Himmelstjerna, J. Krücken, J.E. Hodgkinson

**Affiliations:** a Institute of Infection and Global Health, University of Liverpool, Liverpool Science Park IC2, Brownlow Hill, Liverpool, United Kingdom; b Moredun Research Institute, Pentlands Science Park, Bush Loan, Midlothian, Scotland, United Kingdom; c The Donkey Sanctuary, Sidmouth, Devon, United Kingdom; d Toxalim (Research Centre in Food Toxicology), Université de Toulouse, INRA, ENVT, INP-Purpan, UPS, Toulouse, France; e Institute for Parasitology and Tropical Veterinary Medicine, Freie Universität Berlin, Berlin, Germany

**Keywords:** Anthelmintic resistance, Cyathostomins, Ivermectin, P-glycoproteins

## Abstract

Anthelmintic resistance is a global problem that threatens sustainable control of the equine gastrointestinal cyathostomins (Phylum Nematoda; Superfamily Strongyloidea). Of the three novel anthelmintic classes that have reached the veterinary market in the last decade, none are currently licenced in horses, hence current control regimens focus on prolonging the useful lifespan of licenced anthelmintics. This approach would be facilitated by knowledge of the resistance mechanisms to the most widely used anthelmintics, the macrocyclic lactones (ML). There are no data regarding resistance mechanisms to MLs in cyathostomins, although in other parasitic nematodes, the ABC transporters, P-glycoproteins (P-gps), have been implicated in playing an important role. Here, we tested the hypothesis that P-gps are, at least in part, responsible for reduced sensitivity to the ML ivermectin (IVM) in cyathostomins; first, by measuring transcript levels of *pgp-9* in IVM resistant *versus* IVM sensitive third stage larvae (L3) pre-and post-IVM exposure *in vitro*. We then tested the effect of a range of P-gp inhibitors on the effect of IVM against the same populations of L3 using the *in vitro* larval development test (LDT) and larval migration inhibition test (LMIT). We demonstrated that, not only was *pgp-9* transcription significantly increased in IVM resistant compared to IVM sensitive L3 after anthelmintic exposure (p < 0.001), but inhibition of P-gp activity significantly increased sensitivity of the larvae to IVM *in vitro,* an effect only observed in the IVM resistant larvae in the LMIT. These data strongly implicate a role for P-gps in IVM resistance in cyathostomins. Importantly, this raises the possibility that P-gp inhibitor-IVM combination treatments might be used *in vivo* to increase the effectiveness of IVM against cyathostomins in Equidae.

## Highlights

• *Pgp-9* transcript levels were higher in ivermectin resistant *versus* susceptible cyathostomin populations.
• P-gp inhibitors increased ivermectin effect against cyathostomins *in vitro*.
• P-gp activity may play a role in ivermectin resistance in cyathostomins.

## Graphical abstract

**Figure:**
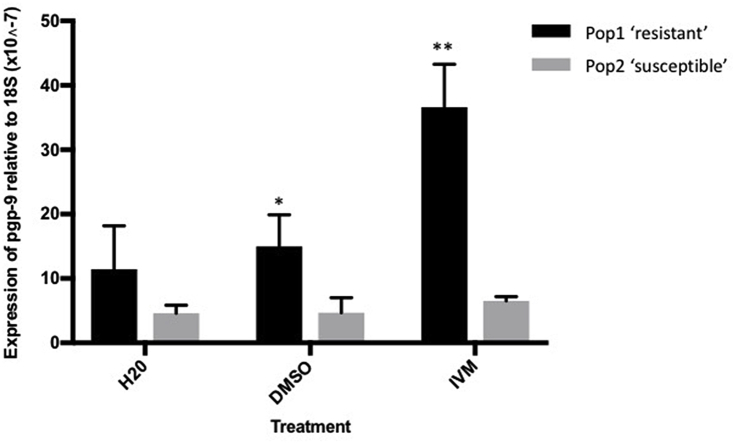

## 1 Introduction

The most prevalent and pathogenic gastrointestinal nematodes (GI) of Equidae in developed countries are the cyathostomins (Love et al., 1999, Kaplan and Vidyashankar, 2012); a group of clade V nematodes belonging to the superfamily Strongyloidea (Lichtenfels et al., 1998). Their pathogenic effect ranges from non-specific weight loss to colic and larval cyathostominosis. The latter is a potentially fatal colitis caused by mass emergence of larvae from the large intestinal wall (Uhlinger, 1991, Murphy and Love, 1997, Lyons et al., 2000, Peregrine et al., 2006). Of the three major classes of anthelmintics available to treat cyathostomin infections, there is already widespread resistance to the benzimadazoles and pyrantel (Nielsen et al., 2014, Peregrine et al., 2014). As a consequence, the macrocyclic lactone (ML) anthelmintics, ivermectin (IVM) and moxidectin (MOX) have been the mainstay of treatment and control programmes in recent years. However, there is now increasing evidence that cyathostomins are developing resistance to MLs (Trawford et al., 2005, Molento et al., 2008, Milillo et al., 2009, Traversa et al., 2009, Trawford and Burden, 2009, Nareaho et al., 2011, Canever et al., 2013, Relf et al., 2014, Tzelos et al., 2017), primarily evidenced by reduced strongyle egg reappearance periods (ERPs) after treatment. Crucially, none of the novel anthelmintics which have reached the animal health market in the last decade, for example, monepantel, derquantel and emodepside, are licenced for use in equids, nor are they likely to be in the near future. For these reasons, the focus of current control regimens is on prolonging the useful life span of the currently effective anthelmintics, in particular ML products (Matthews, 2014). This would be greatly facilitated by an understanding of resistance mechanisms to MLs in cyathostomins, of which little is currently known.

There has been extensive investigation into mechanisms underlying ML resistance in other parasitic nematode species; however, a monogenic mechanism, for example a target site mutation in a glutamate gated chloride channel, has yet to be identified (El-Abdellati et al., 2011, Williamson et al., 2011). One group of genes, those that encode ATP-binding cassette (ABC) transporters, have been strongly implicated in ML resistance in several nematode species (Xu et al., 1998, Ardelli and Prichard, 2007, Lifschitz et al., 2010a, Lifschitz et al., 2010b, Bourguinat et al., 2011, Dicker et al., 2011, Geary et al., 2011, Williamson et al., 2011; Ardelli, 2013, Janssen et al., 2013a, Janssen et al., 2013b, AlGusbi et al., 2014, Tyden et al., 2014, Raza et al., 2015). ABC transporters are large transmembrane proteins found in cells throughout Archaea, Eubacteria and Eukarya, and amongst multiple functions, they are responsible for ATP-dependant efflux of xenobiotic compounds from cells, and so play an important role in protection against toxic environmental compounds (Dassa, 2003). In mammals, through their expression at epithelial barriers such as the blood brain barrier, hepato- and enterocytes, ABC transporters are important in the pharmacokinetic dynamics of hydrophobic drugs such as MLs (Schinkel et al., 1994, Laffont et al., 2002, Ballent et al., 2006, Kiki-Mvouaka et al., 2010).

Numerous ABC transporters have been shown to be expressed in nematodes; for example, *Caenorhabditis elegans* has 14 P-glycoprotein (P-gp) and 8 membrane resistance protein (MRP) genes (Ardelli, 2013). In *H. contortus*, P-gp expression has been shown to predominate in the gastrointestinal (GI) tract, particularly the pharynx and anterior intestine (Smith and Prichard, 2002), leading to the hypothesis that P-gps may play an important role in nematode drug metabolism. In support of this, it was demonstrated that MLs are good substrates for ABC transporters (Lespine et al., 2006, Lespine et al., 2007, Lespine et al., 2009, Dupuy et al., 2006, Dupuy et al., 2010, Kiki-Mvouaka et al., 2010, David et al., 2016), and studies in *C. elegans* using strains with loss-of-function mutations in ABC transporters have shown that various ABC-transporter knockout combinations confer increased sensitivity to MLs (Ardelli and Prichard, 2013, Janssen et al., 2013b). There is growing evidence that ABC transporters, in particular P-gps, are involved in nematode ML resistance. A reduction in P-gp gene heterozygosity after IVM exposure in *O. volvulus* and *H. contortus* indicated that certain P-gp genotypes may confer an advantage for nematodes in the presence of IVM (Ardelli et al., 2005, Ardelli and Prichard, 2007, Blackhall et al., 2008). Up-regulation in P-gp and MRP mRNA was observed in ML resistant strains of *C. elegans* (MRP-1, MRP-6, *pgp-1* and *pgp-2*), *H. contortus* (*pgp-2, pgp-9)* and *T. circumcincta* (*pgp-9)* (James and Davey, 2009, Dicker et al., 2011, Williamson et al., 2011, Ardelli and Prichard, 2013), indicating that increased drug efflux via these channels may play a role in resistance. In *Cooperia oncophora, Ostertagia ostertagi, C. elegans, H. contortus* and *T. circumcincta*, it was shown that addition of P-gp inhibitors to ML resistant nematodes *in vitro* has the effect of increasing ML sensitivity (Bartley et al., 2009, Ardelli and Prichard, 2013, Demeler et al., 2013, AlGusbi et al., 2014, Raza et al., 2015, Ménez et al., 2016). This effect has also been reproduced *in vivo* where a combination of IVM and/or moxidectin with the P-gp inhibitor loperamide increased the effectiveness of the anthelmintics in ML resistant nematode populations in sheep and cattle (Lifschitz et al., 2010a, Lifschitz et al., 2010b). This effect is thought to be due, in part, to increased bioavailability of IVM due to modulation of host P-gps, but there may be a direct effect of P-gp inhibitors on parasite drug transport.

There are little data regarding ABC transporters in cyathostomins; characterisation of these molecules in this group of parasites is complicated by the number of species that exist and that they virtually always occur as co-infections with multiple species (Ogbourne, 1976, Reinemeyer et al., 1984, Love and Duncan, 1992, Bucknell et al., 1995, Gawor, 1995, Traversa et al., 2010). One publication reported partial nucleotide sequences of two P-gp nucleotide-binding domains in several common species of cyathostomins (Drogemuller et al., 2004). Analysis of these sequences suggested the possibility of at least two P-gp genes in the study samples. Subsequently, the full DNA sequence of the *pgp-9* gene in *Cylicocyclus elongatus* was published and in this study, IVM was shown to inhibit *pgp-9* mediated protection of yeast cells against the fungicide ketoconazole (Kaschny et al., 2015). In the current study, the role of *pgp-9* in cyathostomin resistance to IVM was investigated. A real-time PCR assay was used to compare *pgp-9* transcript levels between cyathostomins from: 1) a population of equids with a long history of ML use and reduced strongyle ERP and 2) an equid population that had never been exposed to anthelmintics. The effect of a range of P-gp inhibitors on IVM efficacy was also compared *in vitro* in these nematode populations using the larval development test (LDT) and the larval migration inhibition test (LMIT) (Demeler et al., 2010b, McArthur et al., 2015).

## 2 Materials and methods

### 2.1 Parasite populations

Cyathostomins were sourced from two populations of differing ML sensitivity, Population 1 (Pop 1, IVM-resistant), comprised ‘resistant’ cyathostomins, from donkeys at the Donkey Sanctuary, (Sidmouth, Devon UK) where there was a history of resistance to MLs (Trawford et al., 2005, Trawford and Burden, 2009). ‘Resistant’ cyathostomins were defined by their response to treatment *in vivo,* and were obtained from animals with a faecal egg count (FEC) of ≥500 eggs per gram (epg) within five weeks of administration of IVM or MOX. Population 2 (Pop 2, IVM-naive), were deemed to be ML ‘sensitive’ cyathostomins, and were derived from Konik horses used for conservation purposes by the National Trust (Wicken Fen, East Anglia, UK). This was a closed herd that had not previously received anthelmintics, and additionally IVM median effective concentration (EC-50) values from this cyathostomin population have previously been shown to be significantly lower than those from the Population 1 (McArthur et al., 2015). It was not possible to perform a faecal egg count reduction test (FECRT) to confirm sensitivity in Pop 2 as anthelmintic treatment was prohibited. An archive of morphologically identified adult cyathostomins collected from horses and donkeys post-mortem and stored in ethanol at -20 °C, was used for amplification of *pgp-9* and design of a real-time polymerase chain reaction (PCR) (Hodgkinson et al., 2008).

#### 2.1.1 Strongyle egg extraction

Fresh faeces were placed in air tight polythene bags (Fisher, UK) and strongyle eggs extracted within 1 h of collection or, if not, stored anaerobically in tap water (H_2_O) at room temperature for no longer than one week (Coles et al., 1992). Extracted eggs were decanted into 50 ml containers (VWR, UK) and concentration ascertained by counting the number of eggs in 5 × 10 μl at 100 × magnification (Leica, Stereo). Mean counts were multiplied by 100 to give number of eggs/1 ml. Egg suspensions were used in the LDT immediately after extraction. A subset of eggs from each sample was cultured to third stage larvae (L3) to rule out presence of *Strongylus* spp., and hence ensure that only cyathostomin L3 were used in *in vitro* tests (Thienpont et al., 1986).

#### 2.1.2 Larval culture

Larval culture was performed as described by McArthur et al. (2015) to harvest L3. The concentration of L3 was ascertained by counting the number of L3 in 5 × 10 μl at 100 × magnification and taking an average of each count × 100 to give the number of L3/1 ml. Samples were examined for the presence of *Strongylus* spp. as above, and only samples where no large strongyles were detected were used. Samples were stored at 4 °C and used within eight weeks.

### 2.2 Design of real time PCR for measurement of cyathostomin *pgp-9* transcription

#### 2.2.1 Identification of *pgp-9* DNA sequence from adult cyathostomins

RNA was extracted from groups of 10 adult worms from a mixed adult cyathostomin sample taken from a donkey (W) at the Donkey Sanctuary (DSW), using the QIAGEN RNeasy Mini kit using a bead beater for 2 min at 4800 rpm. cDNA synthesis was performed using the QIAGEN Quantitect Reverse Transcriptase kit and cDNA stored at -20 °C. Partial *pgp-9* translated amino acid sequence from *T. circumcincta* (AN:CBX21126.1) and *H. contortus* (AN:AFX93750.1), and full *pgp-9* translated amino acid sequence from *C. elegans* (AN:CAB03973) and *C. elongatus* (AN: KJ701410) were aligned using Clustal Omega (http://www.ebi.ac.uk/Tools/msa/clustalo/) to identify conserved regions in the nematode *pgp-9* gene; specificity of this region was confirmed by BLAST analysis (http://blast.ncbi.nlm.nih.gov/Blast.cgi?PAGE=Proteins). NCBI primer design (http://www.ncbi.nlm.nih.gov/tools/primer-blast/) with the *C. elongatus* nucleotide sequence (A.N. KJ701410.1) was used to identify a forward primer located in this conserved region, and a reverse primer located in the highly conserved first nucleotide binding domain (Kaschny et al., 2015). Next, degenerate primers were designed by alignment with *H. contortus* (A.N. JX430937.1) nucleotide sequence, thus maximising the likelihood that the primers would amplify *pgp-9* from multiple cyathostomin species. The primers were as follows: forward, PGP9F 5′-CKGCMACRATACAGGCAATR-3′ and reverse PGP9R 5′-GTCCTCACGTCCMGAMARTTG-3. PCR was performed using QIAGEN Taq PCR Master Mix Kit. To each reaction, 25 μl Master Mix, 2.5 μl forward and reverse primer (final concentration 0.5 μM), 18 μl DNase free water, and 2 μl template cDNA were added, to give a total volume of 50 μl. The PCR reaction was run on the Biometra T3 Thermocycler under the following conditions: 3 min at 94 °C, followed by 34 cycles of 1 min denaturation at 94 °C, 1 min at 52 °C and 1 min extension at 72 °C, and a final extension phase of 10 min at 72 °C. PCR products were subjected to electrophoresis on 1% agarose gels at 100 V for 30 min and the products stained for UV visualisation using ‘SYBR safe’ stain (Life Technologies, UK), alongside a 1000 bp ladder (Invitrogen, UK). PCR products were purified using the QIAGEN QIAquick Gel extraction kit, quantified using the Invitrogen Qubit Assay, and cloned using the pGEM-T Easy Vector System (Promega). Plasmid DNA was purified from the cells using the Promega Wizard Plus SV Minipreps DNA Purification System, as per manufacturer's instructions. DNA was quantified using the Qubit assay (Invitrogen) and 100 ng/μl from 10 separate colonies sequenced using a commercial service (Source Biosciences, UK). Resultant sequences (designated DSW A1-5 and B1-5) were entered into NCBI nucleotide BLAST (https://blast.ncbi.nlm.nih.gov/Blast.cgi?PAGE_TYPE=BlastSearch). Alignments between cDNA and amino acid sequences of all clones and *C. elongatus pgp-9* (A.N. KJ701410.1) were created using Clustal Omega, and percentage identity calculated. A maximum likelihood tree with 500 bootstraps using a kimura 2-parameter model, was constructed comparing DW1 A1-5 and B1-5, *C. elongatus pgp-9* (A.N. KJ701410.1), *H. contortus pgp-9* (A.N. JX430937.1)*, T. circumcincta pgp-9* (A.N. FR691848.1)*, C. elegans pgp-9* (C47A10.1 wormbase i.d.) and *Caenorhabditis briggsae pgp-9* (XM_002638567.1) created using MEGA version 6.06 software.

#### 2.2.2 Design and optimisation of a real-time PCR assay for quantification of cyathostomin *pgp-9* cDNA

A SYBR^®^ green (Life Technologies) real time quantitative PCR assay was designed to determine *pgp-9* transcript levels in cDNA samples. A single housekeeping gene, the 18S rRNA gene, was used to allow for multi-species referencing. The 18S assay was designed using a consensus sequence of 99 bp produced from the alignment of the 18S rRNA from six cyathostomin species and other organisms as follows: *C. labiatum* (A.N. AJ223727); *C. ashworthi* (A.N. AJ223346); *C. nassatus,* (A.N. AJ223348); *C. elegans,* (A.N. X03680); *Ascaris suum*, (A.N. U94367.1); *Toxoplasma gondii,* (A.N. L37415); *Schizosaccharomyces pombe,* (A.N. AY251644); *Dirofilaria immitis,* (A.N. AF036638); *Xenopus laevis,* (A.N. X02995); *Drosophila melanogaster,* (A.N. M21017); *Strongyloides stercoralis,* (A.N. M84229); and *Enoplus brevis,* (A.N. U88336). Primers were as follows; 18SRTf 5′ GATTGATTCTGTCAGCGCTATA 3′ and 18SRTr 5′ TAATGAGCCGTTCGCAGT 3’. A standard consensus synthetic 99 bp 18S template corresponding to the cyathostomin species above, was manufactured by Sigma-Aldrich, UK. For *pgp-9,* primers were designed using Primer 3 version 4.0.0 (http://primer3.ut.ee/) based on the sequence data from DSW A1. The primers with the best thermokinetic parameters were chosen and aligned with all 10 sequenced *pgp-9* sequences and *C. elongatus pgp-9* (A.N. KJ701410.1), degenerate primers were designed to maximise amplification of *pgp-9* from multiple cyathostomin species. The degenerate primers for *pgp-9* were PGP9RTF 5′ AYATTGGGCTYGGTCTTGCT 3′ and PGP9RTR 5′ ACCGTTCCYCCTTTCATCGT 3’. The 112 bp sequence including the two primers was synthesised by Sigma-Aldrich, UK, for use as a *Pgp-9* standard with the most commonly occurring base incorporated at each of the 12 sites of nucleotide variation. A 10-fold dilution series of the 18S and *pgp-9* standard oligonucleotides was made in 100 μg/ml yeast tRNA (Invitrogen, UK). Final theoretical copy numbers for each dilution were 5 × 10^−1^, 5 × 10^0^, 5 × 10^1^, 5 × 10^2^, 5 × 10^3^, 5 × 10^4^, 5 × 10^5^, 5 × 10^6^, 5 × 10^7^, 5 × 10^8^. Primers 18SRTf, 18SRTr, PGP9RTF and PGP9RTR were diluted to 100 μM. A master mix was made up with 10 μl 2 × SensiMix (dT) (Bioline, UK), 7 μl DNase/RNase free dH_2_O (Sigma-Aldrich, UK), 1 μl 4 μM forward and reverse primer (either for 18S or *pgp-9*) per sample. To each well of a 96-well plate, 19 μl mastermix were added, followed by 1 μl of each standard concentration. Duplicates were run for each standard concentration and a non-template control (dH_2_O) was included. The plate was run on a DNA Engine Opticon 2 Continuous Fluorescence detector (BioRad), with the reaction conditions: 95° C for 10 min, followed by 35 cycles of 95 °C for 15 s, 53 °C for 30 s and 72 °C for 15 s. The standard curve was repeated with the addition of serial dilutions of cDNA from DSW. Analysis of cDNA concentration, showed that *pgp-9* detection was best at a concentration of 4 ng/μl. The optimised real time PCR was also run on cDNA from *Cylicocyclus nassatus, Cyathostomum pateratum* and *Cyathostomum catinatum* parasites to ensure that the assay could detect *pgp-9* from a range of cyathostomin species (data not presented).

### 2.3 Evaluation of *pgp-9* transcript levels after ivermectin exposure of third stage larvae from mixed species cyathostomin populations of differing ivermectin sensitivity

A total of ∼800,000 and ∼1.1 million L3 were obtained from faeces of 10 animals for each population. L3 were pooled for each population and suspended in 450 ml distilled water (dH_2_O) (Sigma-Aldrich). These were mixed and divided into three aliquots of 50 ml (negative controls), and 6 aliquots of 48.5 ml (test samples) and placed into 50 ml centrifuge tubes. L3 in each aliquot were ex-sheathed with 7% sodium hypochorite (Miltons) for 3.5 min then washed three times by centrifugation at 200 × *g* for 2 min. To three of the 48.5 ml test samples, 1.5 ml DMSO (Sigma-Aldrich, UK) were added to serve as a control for the drug solvent. To the remaining samples, 1.5 ml 2.28 μM IVM (Sigma-Aldrich, UK) in DMSO were added, giving a final IVM concentration of 0.0684 μM. This concentration was chosen as a ‘sub-lethal’ dose (i.e. where percentage larval migration began to decrease, but the majority of the L3 were still alive and presumably still capable of transcribing RNA), based on data from pilot experiments (data not shown). Aliquots were incubated with L3 for 2 h at 26 °C then centrifuged for 2 min at 200 × *g*, the supernatant removed and L3 re-suspended in 20 ml dH_2_O. This was repeated three times. Finally, L3 were transferred to a micro-centrifuge tube and centrifuged for 5 min at 11,000 × *g*. The remaining supernatant was removed and L3 were air dried, re-suspended in 100% ethanol (Sigma-Aldrich, UK) and stored at -20 °C. RNA extraction and cDNA synthesis were carried out on each L3 sample as described above. In the cDNA synthesis step, reverse transcriptase-free negative controls were included for each aliquot. The cDNA concentration in each sample was quantified using the Picogreen DNA quantification kit (Promega). The cDNA concentration in each aliquot was standardised to 2 ng/μl using RNase/DNase-free water (Sigma-Aldrich, UK). Real time PCR for 18S and *pgp-9* was performed alongside standard curves for all samples according to the method above except, to maintain cDNA concentrations close to the optimum described above, 2 μl cDNA were added to each reaction to give a total of 4 ng reverse-transcribed RNA per reaction. Technical duplicates were included for each sample, as well as reverse transcriptase-free negative controls and non-template controls. For each incubation (i.e. dH_2_O, 3% DMSO or 0.15 μg/ml IVM), there were two technical (within assay) repeats in addition to the three biological repeats described above.

### 2.4 Analysis of real time PCR data

Raw data from the real-time PCR was evaluated using Opticon Monitor 3 version 3.1.32.0 software. Standard graph correlations were examined and melting curves observed for each sample; those that did not have a single peak at the expected temperature were excluded from further analysis. Raw data from the qPCR reactions were entered into a Microsoft Excel spreadsheet. The percentage genomic contamination in each sample was calculated from the reverse transcriptase negative control for the raw 18S data; this was used to correct the raw data for 18S and *pgp-9* so that genomic DNA was not counted. The average copy number between each technical repeat was taken for each sample for 18S and *pgp-9*, and the relative transcript level of *pgp-9 gene* compared to the 18S gene, calculated for each biological repeat. These data were analysed with two-way analysis of variance (ANOVA), to compare *pgp-9* transcription between treatments and equid populations, using R statistical software (R Core Team, 2014).

### 2.5 Pharmaceutical preparations for use in the larval development test and larval migration inhibition test

IVM (Sigma-Aldrich, UK) was dissolved in DMSO (Sigma-Aldrich, UK) to give stock solutions of 5000 nM for the LDT and 3420 μM for the LMIT. These were serially diluted in DMSO before use to give final concentrations in the two tests: 25, 13, 6.3, 3.1, 1.6, 0.78, 0.39, 0.20, 0.10, 0.05 nM in the LDT and 102.6, 51.3, 20.5, 10.3, 2.05, 0.684, 0.171, 0.0684, 0.0342, 0.0171 μM in the LMIT. The concentration of the three P-gp inhibitors tested, pluronic 85 (P85), ketoconazole (K) and ivermectin aglycone (IVM-AG), were optimised for use in the LDT and LMIT, so that concentrations were optimal for P-gp inhibitory activity, without having an anthelmintic effect. The concentrations of P85 and ketoconazole that lead to maximum P-gp inhibition had been reported previously as 22 μM and 10 μM, respectively (Bartley et al., 2009). Adaptations of the LMIT and LDT were performed with dose titrations of all three P-gp inhibitors. There was no effect on larval migration or development at the published optimal concentrations for P85 and K, hence they were used in further tests. The concentration of IVM-AG that leads to maximum P-gp inhibition has not been previously established, thus an additional test was run, where increasing concentrations of IVM-AG were compared with increasing concentrations of IVM-AG plus 1 μM IVM. This was done to determine the concentration at which the addition of IVM-AG optimally improved inhibition of larval migration or development compared to IVM alone. The optimal concentration was found to be 60 nM for the LMIT (data not shown). In the LDT, IVM-AG showed anthelmintic activity at concentrations greater than 10 nM so it was necessary to use a concentration of 8 nM, even though this may not have been optimal for P-gp inhibition. K (Sigma-Aldrich, UK) was used at 10 μM in all tests with the LDT and LMIT. For the LDT, a stock solution of 2000 μM in DMSO was used and added in a 0.5:100 ratio to the test, and for the LMIT a 1000 μM in DMSO was used and added in a 1:100 ratio at each stage of the test. Pluronic 85 (P85) (BASF, USA) was used at 22 μM in the LDT and LMIT. For the LDT, a stock solution of 4400 μM in dH_2_O (Sigma-Aldrich, UK) was added in a 0.5:100 ratio. For the LMIT, a stock solution of 2200 μM in dH_2_O was used and added in a 1:100 ratio at each stage of the test. IVM-AG (synthesised at Toxalim, INRA) was used at 8 nM in the LDT and, for this, a stock solution of 1600 nM was used and added in a 0.5:100 ratio. In the LMIT, IVM-AG was used at a concentration of 60 nM and, for this, a stock solution of 6000 nM was used, which was added in a 1:100 ratio.

### 2.6 The larval development test with pluronic 85, ketoconazole and ivermectin aglycone

The LDT with IVM was performed on samples from Pop 1 and Pop 2, as previously described (Demeler et al., 2010b), using the working concentrations of IVM described above in duplicate. Duplicate negative controls of dH_2_O were also included. The LDT was repeated with the addition of 10 μM ketoconazole, 22 μM P85 and 8 nM IVM-AG in all wells, including the negative control, for two samples from each population. The working concentrations of IVM were adjusted to keep the DMSO concentration constant at 0.5%.

### 2.7 The larval migration inhibition test with pluronic 85, ketoconazole and ivermectin aglycone

The LMIT with IVM was performed on samples from Pop 1 and Pop 2, as previously described (McArthur et al., 2015), using the working concentrations of IVM described above. The test was repeated in four parasite samples from each population. For each sample, the test was then repeated with the addition of 10 μM ketoconazole, 22 μM P85 or 60 nM IVM-AG in all wells, including the negative control. The working concentrations of IVM were adjusted to keep the DMSO concentration constant at 3%.

### 2.8 Analysis of dose response data from the larval development test and larval migration inhibition tests

The percentage of larvae which did not develop or migrate in negative controls in each test was defined as the ‘natural mortality’. The raw data were subsequently corrected for ‘natural mortality’ at each concentration and the percentage egg hatch or larval migration re-calculated. The corrected data were used to calculate logistic sigmoid dose response curves in Graphpad Prism 7. For each test, four parameters were defined in a model according to the equation:

y = bottom+(top-bottom)/1 + 10^(logEC−50-x)∗hillslope^

where x = log_10_ concentration, y = percentage hatch/migration, bottom = the y value at the bottom plateau, top = the y value at the top plateau, and hillslope = the gradient of the curve. This enabled calculation of the median effective concentration (EC-50) for comparison between tests. Goodness of fit for each model was evaluated by the R^2^ value and the confidence intervals. Where appropriate parameters (top and bottom) were constrained to allow statistical comparison between repeats using the sum of squares F test, the p value was corrected for multiple comparisons between repeats, with p < 0.008 indicating a significant difference between models.

The ratio between the EC-50 for IVM alone and IVM plus P-gp inhibitor was calculated to give a ‘synergy ratio’ (SR) representing the effect of the inhibitor on IVM efficacy. In addition, the ratio of the EC-50s between the two populations was calculated for the LMIT and LDT to give a resistance ratio representing the difference in IVM sensitivity between the two populations.

## 3 Results

### 3.1 Identification of cyathostomin *pgp-9* sequences

**Fig. 1:**
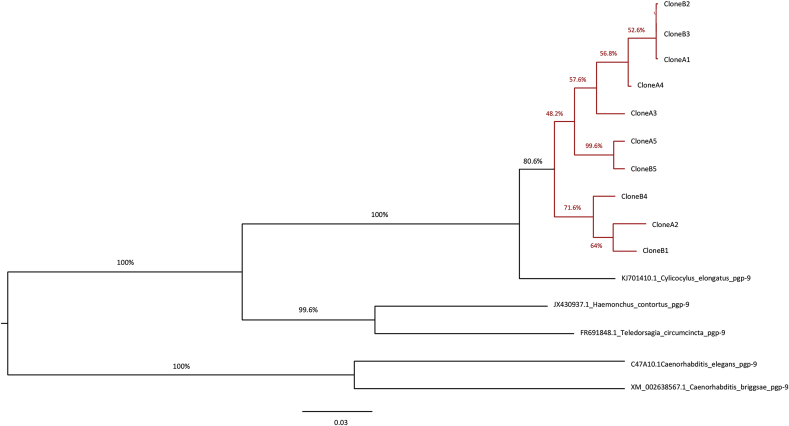
Maximum likelihood phylogenetic tree demonstrating the relationship between nucleotide sequences for DSW (A1-5 and B1-5) (highlighted red) and published *pgp-9* sequences from *Cylicocylus elongatus pgp-9* (A.N. KJ701410.1), *Haemonchus contortus pgp-9* (A.N. JX430937.1)*, Teladorsagia circumcincta pgp-9* (A.N. FR691848.1)*, Caenorhabditis elegans pgp-9* (C47A10.1 wormbase i.d.) and *Caenorhabditis briggsae pgp-9* (XM_002638567.1). (For interpretation of the references to colour in this figure legend, the reader is referred to the web version of this article.)

PCR for *pgp-9* using primers designed from a consensus sequence of *C. elongatus* and *H. contortus* was positive in DSW with an amplicon of the expected size, 1100bp, observed. The nucleotide sequence of clone A1 was found to have 92% identity to the *pgp-9* genes previously identified in *C. elongatus* across 100% of the amplicon. BLASTn analysis showed 77% and 74% identity to *H. contortus* and *T. circumcincta pgp-9,* respectively, and lower levels of identity to other nematode species (Table 1). A nucleotide sequence alignment of all 10 sequences (A1-5 and B1-5) from the DSW *Pgp-9* PCR product showed the range of identity to be between 91.1% (between *C. elongatus* and sequence B4 and A4) and 99.8% (between sequence B2 and B3). The maximum likelihood phylogenetic tree for nucleotide sequences (Fig. 1) indicated with a 100% bootstrap probability that the sequences from DSW are more closely related to *C. elongatus* than to sequences from other nematode species. For the 10 sequences from DSW, there was an 80% bootstrap probability that these constitute a single cluster forming a sister group to *C. elongatus pgp-9*. Within this cluster, there were few differences between sequences and it was unlikely that these represented phylogenetically distinct sequences from different species, as reflected by the lower bootstrap values. The alignment of deduced amino acid coding sequences for the 10 DSW sequences and the corresponding gene region of *C. elongatus*, is shown in Supplementary Fig. 1. The sequence revealed the typical domain arrangement of one half of the P-gp gene with an ABC transporter transmembrane domain containing transmembrane helices followed by the first part of the first nucleotide binding domain containing the highly-conserved Walker A/P loop (CDD accession number cd03249).

**Table 1:** Results of NCBI blastn search of the sequence of DSW A1 *pgp-9* PCR product.

Nucleotide sequence hit	Accession	Max Score	Total Score	Query cover	E-value	Identity
*Cyclocylcus elongatus* P-glyocprotein (pgp9) mRNA, complete cds	KJ701410.1	1418	1418	100%	0.0	92%
*Haemonchus contortus* isolate Weybridge P-glycoprotein 9a mRNA partial cds	JX430937.1	778	778	99%	0.0	77%
*Teladorsagia circumcincta* partial mRNA for p-glycoprotein 9, nucleotide binding domain 1 (pgp-9 gene)	FR691848.1	677	677	99%	0.0	74%
*Necator americanus* ABC transporter transmembrane region mRNA	XM_013451424.1	333	586	79%	3e-88	80%
*Caenorhabditis briggsae* C CBR-PGP-9 protein (Cbr-pgp-9) mRNA complete cds	XM_002638567.1	284	284	89%	1e-73	67%
*Caenorhabditis remanei* hypothetical protein (CRE_22140) mRNA, complete cds	XM_003091578.1	269	269	92%	3e-69	66%
*Caenorhabditis remanei* hypothetical protein (CRE_27936) mRNA, complete cds	XM_003090002.1	266	266	96%	3e-68	66%
*Cyclostephanus goldi* genome assembly C_goldi_Cheshire, scaffold CGOC_contig0005008	LL376643.1	262	392	41%	4e-67	74%
*Cyclostephanus goldi* genome assembly C_goldi_Cheshire, scaffold CGOC_contig0000757	LL362081.1	255	1051	88%	6e-65	75%
*Cyclostephanus goldi* genome assembly C_goldi_Cheshire, scaffold CGOC_contig0002917	LL371363.1	230	580	58%	2e-57	90%

### 3.2 *Pgp-9* mRNA expression in L3 from two mixed species populations of cyathostomins with differing ivermectin sensitivity

**Fig. 2:**
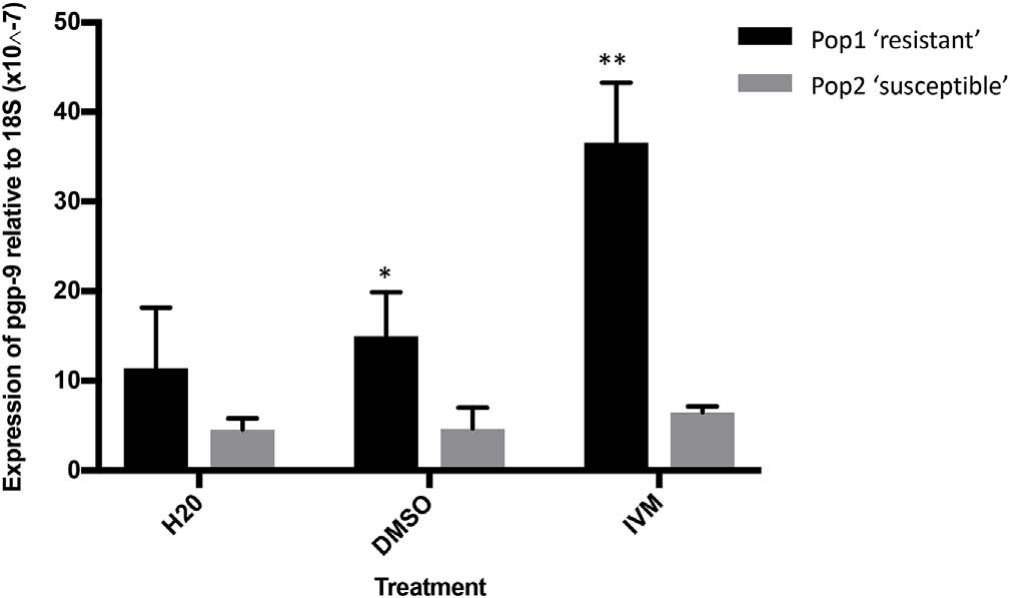
Bar chart showing transcript levels of *pgp-9* mRNA relative to the housekeeping gene, 18S, in third stage larvae in Populations 1 ‘resistant’ and 2 ‘susceptible’, which had been incubated with distilled water (H2O), dimethyl sulfoxide (DMSO) or ivermectin (IVM) (*p = <0.05; **p= <0.001). Within each population, there was a significant effect of the different treatments for Pop 1 (p = 0.005), but not for Pop 2 (p = 0.31).

Copy numbers of *pgp-9* transcript relative to the housekeeping gene 18S in L3 in Pop 1 (IVM-resistant) and Pop 2 (IVM-sensitive) after incubation with dH_2_O, 3% DMSO or 0.0684 μM IVM for 2 h are represented in Fig. 2. After exposure to dH_2_O, there was no significant difference in *pgp-9* transcript levels between Pop 1 and Pop 2 (p = 0.159). After exposure to DMSO, there were higher levels of *pgp-9* transcript measured in Pop 1 compared to Pop 2 (p = 0.03). After exposure to IVM there were higher levels of in *pgp-9* transcript measured in Pop 1 compared to Pop 2 (p < 0.001). Within each population, there was a significant effect of the different treatments for Pop 1 (p = 0.005), but not for Pop 2 (p = 0.31). The fold-increase in *pgp-9* mRNA level between the DMSO and IVM treatment in Pop 1 was 2.4.

### 3.3 Evaluating the effect of P-glycoprotein inhibitors on ivermectin efficacy in the larval development test and larval migration inhibition test

**Fig. 3:**
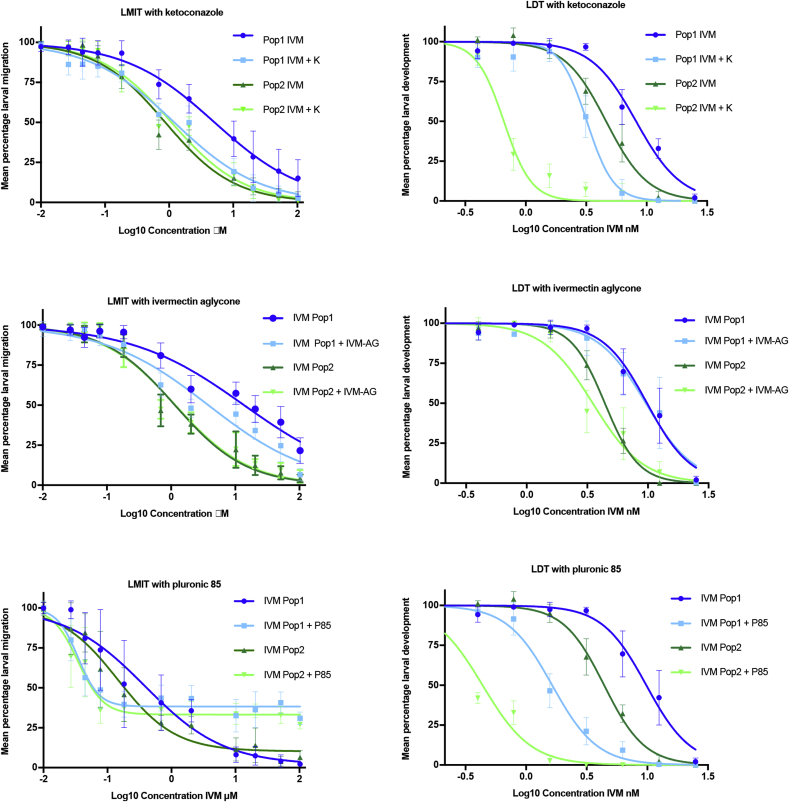
Ivermectin (IVM) concentration response curves in Population 1 ‘resistant’ and 2 ‘susceptible’ (Pop 1, Pop 2) in the larval development test (LDT) and larval migration inhibition test (LMIT), with and without the addition of the P-glycoprotein inhibitors ketoconazole (K), ivermectin aglycone (IVM-AG) and pluronic 85 (P85).

Four-parameter dose response curves for each test (LMIT and LDT) with each P-gp-inhibitor (K, IVM-AG and P85) are shown in Fig. 3. The EC-50 values for the curves and the synergy ratio (SR) for IVM and IVM plus P-gp inhibitor are represented in Table 2. For each group of tests, for example, IVM alone and IVM + P-gp inhibitor in Pop 1 and 2 in the LDT/LMIT, a significant difference was found between the dose response curves, using the sum of squares F test (p < 0.001 in both the LDT and LMIT). From these results, in the LDT, P85 and K reduced EC-50s for IVM in Pops 1 and 2, but to a lesser extent in Pop 1. In the LDT, IVM-AG had no positive effect on IVM efficacy. In the LMIT, IVM-AG and K increased the effect of IVM in Pop 1, but had little effect in Pop 2. In the LMIT, P85 increased the effect of IVM at lower anthelmintic concentrations, but decreased the effect at high concentrations; this had the effect of raising the bottom line of the four-parameter curve which meant that, although an EC-50 value could be calculated for IVM with P85 in the LMIT, it could not be meaningfully compared with IVM alone using an SR. The mean resistance ratio was 2.1 in the LDT and 9.0 in the LMIT.

**Table 2:** EC-50 values calculated for each population in the larval development test (LDT) and larval migration inhibition test (LMIT), with ivermectin (IVM) and IVM plus Pluronic 85 (P85) (22 μM), ketoconazole (K) (10 μM) and ivermectin aglycone (IVM-AG) (8 and 60 nM for the LDT and LMIT, respectively). For each P-glycoprotein (P-gp) inhibitor a ratio of the EC-50 with IVM alone and the EC-50 with P-gp inhibitor is shown, defined as the synergy ratio (SR).

	Assay (LDT or LMIT)	P-gp inhibitor	EC-50 IVM alone, (95% confidence interval)	EC-50 IVM + p-gp inhibitor (95% confidence interval)	Synergy ratio
Population 1	LDT	P85	9.81 (8.66–11.08) nM	1.63 (1.48–1.80 nM	6.0
		K	8.17 (7.44–8.98) nM	3.20 (2.94–3.47) nM	3.6
		IVM-AG	9.82 (8.69–11.08) nM	9.59 (8.18–11.21) nM	1.0
	LMIT	P85^a^	0.38 (0.23–0.72) μM	(0.02 (0.02–0.03) μM)	(19)
		K	5.11 (4.30–6.06) μM	1.20 (1.04–1.40) μM	4.3
		IVM-AG	13.70 (11.03–17.13) μM	3.79 (3.03–4.72) μM	3.6
Population 2	LDT	P85	4.40 (4.07–4.75) nM	0.43 (0.38–0.48) nM	10.2
		K	4.62 (4.23–5.04) nM	0.65 (0.60–0.71) nM	7.1
		IVM-AG	4.40 (4.14–4.68) nM	3.40 (3.14–4.04) nM	1.3
	LMIT	P85^a^	0.14 (0.10–0.20) μM	(0.02 (0.02–0.03) μM)	(7)
		K	0.86 (0.77–0.97) μM	1.08 (0.91–1.26) μM	0.8
		IVM-AG	1.14 (0.95–1.36) μM	1.16 (0.97–1.38) μM	1.0

a As the baseline of the dose response curve differed between IVM and IVM + P85 in the LMIT, the EC-50s cannot be compared with that of IVM alone, and hence the EC-50 and synergy ratio are shown in brackets.

## 4 Discussion

This study demonstrated that *pgp-9* mRNA is upregulated in cyathostomins with phenotypic ML resistance, and that P-gp inhibitors increase the effect of IVM against these ML-resistant cyathostomins in a LMIT. Taken together, this suggests a role for P-gps in IVM resistance in these nematodes, and raises the possibility that P-gp inhibitors could be used in equids, in combination with IVM, to increase efficacy against IVM-resistant cyathostomin populations, as has been done in sheep and cattle (Lifschitz et al., 2010a, Lifschitz et al., 2010b).

In this study, to design a real-time PCR assay to measure *pgp-9* transcript in cyathostomins, *pgp-9* cDNA was amplified from a mixed species cyathostomin population. Preliminary analysis of the data in a maximum likelihood phylogenetic model predicted with 100% certainty that there was a true difference between the sequences defined in this population and the previously characterised *C. elongatus* sequence (A.N. KJ701410.1); this may reflect, either intra-specific variation or, more likely, inter-specific variation due to the presence of multiple different cyathostomins species in the population studies here (Cwiklinski, Matthews and Hodgkinson, unpublished). Additionally, further analyses showed that the novel sequences reported here clustered into two groups, raising the possibility that *pgp-9* sequences from two species of cyathostomin were amplified. Further work is required to measure intra- and inter-specific variation of *pgp-9* sequences across cyathostomin species.

It is possible that selection for particular cyathostomins species with upregulated P-gp transport pathways has occurred in the ‘resistant’ population here; a mechanism which differs from one in which several members of a mixed species population upregulate P-gp in response to selection pressure. However, in support of the latter, data from the ‘resistant’ population shows a range of species present (*Cyathostomum catinatum, Cya. tetracantum, Cya. pateratum, Cylicostephanus longibursatus, Cylicocyclus nassatus*) (Cwiklinski, Matthews and Hodgkinson unpublished). These species are concordant with those common species that have been reported in populations across a range of geographic regions and which have had varying exposure to macrocyclic lactone anthelmintics (Bucknell et al., 1995, Gawor, 1995, Love and Duncan, 1992, Ogbourne, 1976, Reinemeyer et al., 1984, Traversa et al., 2010, Eysker and Pandey, 1989, Getachew et al., 2010, Kharchenko et al., 2009, Krecek et al., 1994).

Interestingly, it was noted that the DMSO solvent also induced a modest increase in *pgp-9* expression in the ‘resistant’ population, but not in the ‘sensitive’ population. As P-gp drug efflux represents a non-specific mechanism designed to remove xenobiotics, it is possible that in a population of parasites which are upregulating this mechanism, exposure to chemicals such as DMSO may elicit a reactive upregulation of P-gp; however, this hypothesis requires further investigation.

The higher levels of *pgp-9* transcript detected in ML resistant *versus* ML sensitive cyathostomins is in agreement with studies in *T. circumcincta, H. contortus* and *Cooperia oncophora* (Dicker et al., 2011, Williamson et al., 2011, Areskog et al., 2013, Godoy et al., 2016). Strong evidence for a role of *pgp-9* in anthelmintic resistance in *T. circumcincta* has recently been reported in a genomic introgression mapping model of field-derived multiple-anthelmintic resistant worms, where a concurrent increase in copy number and transcript levels was found for this gene (Choi et al., 2017). Additionally, Williamson et al. (2011), measured transcript levels in *H. contortus* L3 cultured from multi-drug resistant *versus* susceptible parasites that had been passaged through goats, and found a 2.65-fold difference in *pgp-9* transcript level, similar to that observed here. Dicker et al. (2011) measured transcript levels in all life cycle stages of *T. circumcincta* from a multidrug resistant v*ersus* susceptible isolate, and reported a larger fold-difference (17.49) in *pgp-9*. The reason for the larger difference in transcript level observed in this study in comparison to the current and other findings, may be that the data was generated using a homogenous multi-class resistant strain, with ML FECR values of 60%, whereas here a reduced ERP was used as a proxy for ML resistance. Additionally, the samples used here were likely to encompass several cyathostomin species, which may have introduced variation if the species had different transcript levels.

Different P-gps have been implicated in the transport of ivermectin and ML resistance in other parasitic nematodes; for example, *pgp-11* in *Cooperia oncophora* and *Parascaris equorum* (De Graef et al., 2013, Janssen et al., 2013a). De Graef et al. (2013) demonstrated increased transcript levels of *pgp-11* in adult worms isolated from calves that had been treated with IVM or MOX. In addition, they were able to induce increased transcript levels in L3 by exposing them to IVM *in vitro* in resistant, but not susceptible isolates, similar to the findings here. Studies in *C. elegans* have shown that different combinations of ABC transporter are associated with IVM and MOX sensitivity, indicating that different compounds are linked to specific transporters associated with resistance (James and Davey, 2009, Ardelli and Prichard, 2013). Variations in P-gp subtype associated with resistance probably reflect a difference in functionality of corresponding genes, such that the specific P-gp involved in ML efflux may differ among nematode species and ML sub-type; however, it has been shown that increased susceptibility of *C. elegans* carrying a loss-of-function mutation in the *pgp-11* gene could be rescued by expression of a *Parascaris* sp. *pgp-11* transgene (Janssen et al., 2015), hence further work is required to clarify these complex interactions.

It was demonstrated here that the effect of IVM in ‘resistant’ cyathostomins could be increased *in vitro* by its combination with certain P-gp inhibitors. The LDT and LMIT were chosen as they are established methods for evaluating IVM efficacy in parasitic nematodes (Demeler et al., 2010a, Demeler et al., 2010b, McArthur et al., 2015). The RR between the two cyathostomin populations reported here was lower for the LDT than the LMIT, in agreement with previous studies in cyathostomins where the LDT has shown poor correlation with *in vivo* phenotype (Tandon and Kaplan, 2004, Lind et al., 2005). In *H. contortus*, the LDT is an established technique for detecting differences in drug sensitivity (Dolinska et al., 2013). These contrasting findings in cyathostomins may indicate that they differ with respect to the mode of action of IVM, and hence potential mechanisms of resistance. Overall these data confirm, as previously reported by McArthur et al. (2015), that in cyathostomins the LMIT is a useful *in vitro* tool for comparing the IVM sensitivity of L3 populations.

Addition of P-gp inhibitors impacted the effect of IVM in the LMIT and LDT. This is consistent with studies in other parasitic nematodes (Bartley et al., 2009, Ardelli and Prichard, 2013, Demeler et al., 2013, AlGusbi et al., 2014, Raza et al., 2015). Overall, the effect was dependent on the isolate, the test (and hence the life cycle stage) and the P-gp inhibitor. P-gp inhibitors have been shown to have different modes of action and levels of affinity for different ABC transporters. Ketoconazole (K), an antifungal imidazole used in veterinary medicine, is a potent inhibitor of cytochrome p450 in humans (Maurice et al., 1992) and has been shown to alter the pharmacokinetics of several P-gp substrate drugs by competitive inhibition at P-gp channels (Ward et al., 2004, Kageyama et al., 2005, Hugnet et al., 2007, Bartley et al., 2009, Bartley et al., 2012). Additionally, it has been shown to compete with IVM at the cyathostomin *pgp-9* channel (Kaschny et al., 2015). Ivermectin aglycone (IVM-AG) is an avermectin derivative that should act by competitive inhibition as predicted by in silico docking on the nematode Cel-P-gp1 (David et al., 2016). Its activity is comparable with verapamil, one of the most potent P-gp inhibitors, and it has greater affinity for nematode *versus* mammalian P-gp (Lespine et al., 2013). Pluronic 85 (P85) is a polaxomer with a multimodal action, and is more commonly used as a nanocarrier in drug delivery systems; it incorporates into cell membranes changing their microviscosity, which has the effect of inhibiting ATPase activity, thus reducing activity of ABC transporters (Batrakova and Kabanov, 2008). They have been shown to be potent inhibitors of P-gps and other ABC transporters such as MRP1 and MRP2 (Shaik et al., 2008). Here, in the LMIT, IVM-AG and K increased the effect of IVM on L3 with a ‘resistant’ (Pop 1) phenotype, but had little effect on L3 with a sensitive phenotype (Pop 2). This suggests that P-gp activity is upregulated in the resistant cyathostomin population, which corroborates the finding of increased *pgp-9* transcript in L3 from the same population. A study by Demeler et al. (2013), tested different P-gp inhibitors against *C. oncophora* in the LDT and LMIT, and reported similar results. In the current study, in the LMIT, P85 had a bi-modal effect, whereby it increased the effect of IVM in both populations at low IVM concentrations, and at high IVM concentrations the effect was reversed. One study in *C. elegans* has also shown that the effect of P-gp inhibitors can be dependent on IVM concentration, although there was not a clear reversal of the effect as seen here (Ardelli and Prichard, 2013). On the other hand, a study using P85 as a P-gp inhibitor with IVM in a larval feeding assay in *H. contortus* and *T. circumcincta*, did not report a bimodal effect (Bartley et al., 2009). One point to note is that much higher concentrations are used in the LMIT than in assays on earlier larval stages, and hence the effect seen here may be an interaction specific to these high concentrations.

In the LDT, K and P85 increased the effect of IVM in both cyathostomin populations. As mentioned above, the LDT did not differentiate as well between the resistant and sensitive populations, and therefore it follows logically that it also did not differentiate between the effects of P-gp inhibitors. Demeler et al. (2013) and Bartley et al. (2009) also reported little differential effect of P-gp inhibitors between resistant and sensitive in the LDT and a larval feeding assay respectively. So, although their effect does not appear to be clearly linked with IVM resistance status in the early larval stages of cyathostomins, P-gp inhibitors markedly increase the effect of IVM, which suggests that these stages express P-gps which have the potential to extrude IVM. In contrast to K and P85, it was found that IVM-AG did not have an effect in the LDT. This was most likely due to its use at a sub-optimal concentration for P-gp inhibition (during optimisation it was found that it had an independent effect at higher concentrations). Given its activity in the LMIT, it is likely that it would have a synergistic effect in the LDT if used at a higher concentration, however it was not possible to test this using our experimental set-up.

As observed here, there are reports of differing effects of P-gp inhibitors dependent on parasite population, test and P-gp inhibitor used. For example, Raza et al. (2015) tested P-gp inhibitors, tariquidar, zosuquidar, elacridar, verapamil and valspodar, against *H. contortus* larvae of differing anthelmintic (IVM, levamisole, thiabendazole) sensitivity, in the LDT and LMIT. They found that several compounds increased sensitivity of both resistant and susceptible isolates, for example, tariquidar with IVM in the LMIT and zosuquidar with IVM in the LDT, whereas others had a significant effect on the resistant isolate only. There is also evidence that there are differences between species or isolates; for example, one study investigating the effect of verapamil on IVM in the LMIT showed there to be a positive effect in resistant and sensitive isolates from *Ostertagia ostertagi,* but only in a resistant isolate of *C. oncophora* (AlGusbi et al., 2014). Our data supported by these studies suggest that some inhibitors interact with P-gps representing intrinsic pathways present across nematode populations with different drug sensitivities, and others interact with P-gps of significance only to resistant worms, and may represent an acquired resistance mechanism. These interactions appear to alter depending on the test (and hence life cycle stage) and nematode species/isolate.

Due to the evidence from *in vitro* studies, there is increasing interest in the potential use of P-gp inhibitors to augment treatment efficacy *in vivo*. The proposed mechanisms of such combinations are: (i) reducing efflux of drug from nematodes, and hence increasing the concentration of drug at its target site within the nematode, and (ii) reducing excretion of drugs by host animals, and hence increasing bioavailability. Based on the results here, IVM-AG and ketoconazole are potential candidates for taking forward to trials, as they both increased the effect of IVM in the ‘resistant’ population. To date, the results of trials in other species have been equivocal. One study in sheep showed that, although the plasma concentration of IVM was significantly increased by co-administration with K, there was no effect on efficacy against *H. contortus* as measured by FECRT (Bartley et al., 2012). The same study showed a moderate effect of P85 on IVM efficacy, even though the plasma concentration was similar to that seen with K, which might indicate that P85 had a direct effect on parasite P-gp function. The study that showed the most convincing effect of a P-gp, combined loperamide with IVM in sheep against mixed GI nematode infection and led to a markedly improved efficacy (Lifschitz et al., 2010a). Loperamide has been shown to increase plasma concentration of MLs (Lifschitz et al., 2002) and, additionally it reduces faecal transit time and therefore may increase exposure time of parasites to ML in the gut. Based on the data presented here it would be logical to suggest an *in vivo* trial of Pgp-inhibitor-IVM combination therapy in Equidae, and reassuringly some known P-gp inhibitors have already been used systemically in Equidae, for example K (Nappert et al., 1996), and loperamide (Sanchez, 2014), which may facilitate future work. When considering *in vivo* trials it is important to be aware of the potential side effects of drug interactions, for example increased toxicity of IVM due to increased bioavailability and plasma levels, however, toxicity has not been noted in published *in vivo* trials undertaken in other species.

## 5 Conclusion

The data presented implicate a role for P-gps in reduced sensitivity to IVM in cyathostomins. The *in vitro* data confirm that IVM interacts with P-gps in cyathostomins. Specifically, K and IVM-AG increased the effect of IVM in the resistant cyathostomin isolate to a greater extent than in the sensitive isolate, implicating interference with an acquired resistance mechanism. These data support the possibility that P-gp inhibitors could be used in combination with IVM *in vivo,* as a novel approach to improving the efficacy of IVM in resistant cyathostomin populations.

## Role of funding source

This work was funded by the Donkey Sanctuary UK, Petplan Charitable Trust (grant number 12-55) and The University of Liverpool, Institute for Infection and Global Health. The former was involved in the provision of equid faecal samples for the study.

## Data availability

The research materials supporting this publication can be accessed by contacting the corresponding author at peachey14@hotmail.com.

## Appendix A Supplementary data

Online data Amino acid alignment of translated sequence from a consensus of all DSW sequences (DSW_C) and *Cylicocyclus elongatus pgp-9* amino acid sequence (A.N. AJM87336.1) (C_elongatus_pgp9). Amino acid changes are indicated underneath the alignment, with * indicating conserved,: indicating strongly similar,. indicating weakly similar and blank indicating different. The characteristic regions of ABC transporters are highlighted with text boxes in the two nucleotide binding domains. Colour coding is as follows: red = Walker A/P loop; black = Q loop; yellow = ABC transporter signature motif; green = Walker B; Purple = H loop (CDD A.N. cd03249). Online data
