# Supplementary material for: P-glycoproteins play a role in ivermectin resistance in cyathostomins
Source: Int J Parasitol Drugs Drug Resist. 2017 Oct 25;7(3):388–98. doi: 10.1016/j.ijpddr.2017.10.006 (PMC5681340; doi:10.1016/j.ijpddr.2017.10.006)
Supplement: Online data — Amino acid alignment of translated sequence from a consensus of all DSW sequences (DSW_C) and Cylicocyclus elongatus pgp-9 amino acid sequence (A.N. AJM87336.1) (C_elongatus_pgp9). Amino acid changes are indicated underneath the alignment, with * indicating conserved,: indicating strongly similar,. indicating weakly similar and blank indicating different. The characteristic regions of ABC transporters are highlighted with text boxes in the two nucleotide binding domains. Colour coding is as follows: red = Walker A/P loop; black = Q loop; yellow = ABC transporter signature motif; green = Walker B; Purple = H loop (CDD A.N. cd03249). [file mmc1.pdf]

```
C_elongatus_pgp9      MGLFKKKEEKEKPTISTEGSKGSEEEEEAPKASIVQLFRYASGFDKLLLLLGCLVSIATGVGMPLMSIIMGNVSQNFMDVTGNYTDPNLIHQFEHDVIQNCLKYVYLGCGIFAAATIQAM
DSW_C                 ------------------------------------------------------------------------------------------------------------------------

C_elongatus_pgp9      CFLTVCENLVNQLRREFFKAILRQDITWYDKNNSGTLAPKLFDNLERVKEGTGDKLGLMIQFVAQFFGGFIVAFTYDWKLTLIMMSLSPFMIICGAFIAKLMASAATEEAKKYAVAGGIA
DSW_C                 -----CENLVNQLRREFFKAILRQDITWYDKNNSGTLAPKLFDNLERVKEGTGDKLGLMIQFVAQFFGGFIVAFTYDWKLTLIMMSLSPFMIICGAFIAKLMASAATEEAKKYAVAGGIA
                           *******************************************************************************************************************

C_elongatus_pgp9      EEVLTSMRTVIAFNGQPYECERYDVALAAGRSTGIKKSLYIGLGLALTFTIMFSSYCLAFWVGTDFVYKGTMKGGTVMTVFFSVMMGSMALGQAGPQFAVLGTAMGAAGSLYQIIDREPE
DSW_C                 EEVLTSMRTVIAFNGQPYECERYDVALAAGRSTGIKKSLYIGLGLALTFAIMFSSYCLAFWVGTDFVYNGTMKGGTVMTVFFSVMMGSMALGQAGPQFAVLGTAMGAAGSLYQIIDREPE
                      *************************************************:******************:***************************************************

C_elongatus_pgp9      IDAYLKAGMKPSNLKGRISVSSVKFSYPTRPDIPILKGISFEANPGETIALVGSSGCGKSTIIQLLLRYYDPAGGKISIDGIEIDKINIEYLRNYIAVVSQEPVLFNTTIEQNIRYGRED
DSW_C                 IDAYSTAGMKPSNLRGRISVSSVKFSYPTRPDIPILKGISFEANPGETIALVGSSGCGKSTIIQLLLRYYDPAGGKISIDGVEINKINIEFLRNYI------------------------
                      **** .********:******************************************************************:**:*****:*****

C_elongatus_pgp9      ITEAEIIAALRKANAYNFVQSFPEGIKTNVGDRGTQMSGGQKQRIAIARALVRDPKILLLDEATSALDAESEHVVQQALENASQGRTTIVIAHRLSTIRNADRIIAMKDGEVMEVGTHDE
DSW_C                 ------------------------------------------------------------------------------------------------------------------------

C_elongatus_pgp9      LIARKGLYHELVNAQVFADVDDDMAKGAGKRKSVSSRRSSTSSIGHPELRRLKSQLSQEVDKIEQSDPKKAEKDLERLKKELEEEGAVKANLFKILHYARPEWAFIFIAVLSAIVQGCVF
DSW_C                 ------------------------------------------------------------------------------------------------------------------------

C_elongatus_pgp9      PAFSLFFTEIIEVFARPPGDPNLQSRGHFWALMFLLLGGVEAVCMITQCFFFGLSAERLTMRLRSKVFHNVMRMDAAYFDMPRHSPGKITTRLATDAPNVKSAIDYRFGSVFNSFVSVCC
DSW_C                 ------------------------------------------------------------------------------------------------------------------------

C_elongatus_pgp9      GIGIAFYFGWQMALLTIAIFPLAGVAHGFQMRFMSGRAGGDAKEMENSGKIAMEAIENIRTVQALTLEHRLHHLFCQHLDGPHKTNKRRAIMQGGAYGFSSSIFFFLYAASFRFGLWLIL
DSW_C                 ------------------------------------------------------------------------------------------------------------------------

C_elongatus_pgp9      NGDMMPMNVLRVLFAISFTAGSLGFASAYFPEYVKATFAAGLIFNMLKDEPRIDGMTDKGKKPKLTGSISLKNVFFNYPERPNVPILQGLDVSVQPGETLALVGPSGCGKSTVVSLLERL
DSW_C                 ------------------------------------------------------------------------------------------------------------------------

C_elongatus_pgp9      YDPLDGVVAVDGNDLREMNPTHLRSHIALVSQEPILFDTSIRDNIVYGLPAGSVTEAMIMEVAQRANIHKFISELPDGFNTRVGEKGTQLSGGQKQRIAIARALIRNPKILLLDEATSAL
DSW_C                 ------------------------------------------------------------------------------------------------------------------------

C_elongatus_pgp9      DTESEKLVQEALDKASKGRTCIVVAHRLSTVVNCNCIMVVKSGKIVEKGTHNELMQAKGAYWALTQKQNIHTN
DSW_C                 -------------------------------------------------------------------------
```
